# The Role of Genetic Testing in the Clinical Practice and Research of Early-Onset Parkinsonian Disorders in a Hungarian Cohort: Increasing Challenge in Genetic Counselling, Improving Chances in Stratification for Clinical Trials

**DOI:** 10.3389/fgene.2019.01061

**Published:** 2019-10-31

**Authors:** Anett Illés, Dóra Csabán, Zoltán Grosz, Péter Balicza, András Gézsi, Viktor Molnár, Renáta Bencsik, Anikó Gál, Péter Klivényi, Maria Judit Molnar

**Affiliations:** ^1^Institute of Genomic Medicine and Rare Disorders, Semmelweis University, Budapest, Hungary; ^2^Department of Neurology, University of Szeged, Szeged, Hungary

**Keywords:** Parkinson’s disease, next-generation sequencing, genetic risk, monogenic forms, risk factors, early- onset, Parkinsonism, Parkinsonian disorders

## Abstract

The genetic analysis of early-onset Parkinsonian disorder (EOPD) is part of the clinical diagnostics. Several genes have been implicated in the genetic background of Parkinsonism, which is clinically indistinguishable from idiopathic Parkinson’s disease. The identification of patient’s genotype could support clinical decision-making process and also track and analyse outcomes in a comprehensive fashion. The aim of our study was to analyse the genetic background of EOPD in a Hungarian cohort and to evaluate the clinical usefulness of different genetic investigations. The age of onset was between 25 and 50 years. To identify genetic alterations, multiplex ligation-dependent probe amplification (n = 142), Sanger sequencing of the most common PD-associated genes (n = 142), and next-generation sequencing (n = 54) of 127 genes which were previously associated to neurodegenerative disorders were carried out. The genetic analysis identified several heterozygous damaging substitutions in PD-associated genes (*C19orf12*, *DNAJC6*, *DNAJC13*, *EIF4G1*, *LRRK2*, *PRKN*, *PINK1*, *PLA2G6*, *SYNJ1*). CNVs in *PRKN* and *SNCA* genes were found in five patients. In our cohort, nine previously published genetic risk factors were detected in three genes (*GBA*, *LRRK2*, and *PINK1*). In nine cases, two or three coexisting pathogenic mutations and risk variants were identified. Advances of sequencing technologies make it possible to aid diagnostics of PD by widening the scope of analysis to genes which were previously linked to other neurodegenerative disorders. Our data suggested that rare damaging variants are enriched versus neutral variants, among PD patients in the Hungarian population, which raise the possibility of an oligogenic effect. Heterozygous mutations of multiple recessive genes involved in the same pathway may perturb the molecular process linked to PD pathogenesis. Comprehensive genetic assessment of individual patients can rarely reveal monogenic cause in EOPD, although it may identify the involvement of multiple PD-associated genes in the background of the disease and may facilitate the better understanding of clinically distinct phenocopies. Due to the genetic complexity of the disease, genetic counselling and management is getting more challenging. Clinical geneticist should be prepared for counselling of patients with coexisting disease-causing mutations and susceptibility factors. At the same time, genomic-based stratification has increasing importance in future clinical trials.

## Introduction

Parkinsonian disorders are a group of progressive neurodegenerative movement disorders, in which pathogenesis both genetic and environmental factors play a role. Parkinson’s disease is the most common among them which responsible for approximately 80% of patients with Parkinsonism. The differential diagnosis of Parkinsonism from common idiopathic Parkinson’s disease generally stands challenges for clinicians, because of heterogeneous phenotypes (motor and nonmotor symptoms), related comorbidities, and the absence of specific biomarkers (Klein and Schlossmacher, 2006). Consequently, in this study, the term Parkinsonian disorders (PDs) is used for expressing the different forms of Parkinsonism, which include both Parkinson’s disease and Parkinson-plus syndromes. Until now, several genes were linked to different PD phenotypes, which may provide a new possible tool for clinicians to distinguish the specific patterns of symptoms or disease course. Moreover, it is important to emphasise that, even the similar involvement of these disease-genes, the clinical phenotype can be variable, although it differs considerably from that of idiopathic PD (Klein, 2005). Furthermore, other movement disorders can sometimes mimic PD phenotype, which also could make more difficult to set up the proper diagnosis without an effective aid by biomarkers.

Studies with familial and sporadic cases identified large number of genetic factors, which are usually not ready to be considered for everyday clinical practice. Based on previous studies, the rare, highly penetrant pathogenic mutations result in monogenic familial forms, although sporadic forms are commonly caused by interactions of environmental and genetic risk factors (Karimi-Moghadam et al., 2018). Over the past few years, the high-throughput next-generation sequencing (NGS) technology pave the way to identify the involvement of several candidate genes in the development of PD (Shulskaya et al., 2018). Dysfunctional protein products resulting from rare mutations with functional consequence in these genes are generally involved in the mitochondrial and protein quality control processes, synaptic transmission, and vesicular recycling pathways related to the homeostasis of neurons. Although, more and more knowledge is gathered in the field of the underlying pathogenesis of PD, and extensive testing of candidate genes is in progress, the genetic background still remains unidentified in 40% of all PD-affected cases (Karimi-Moghadam et al., 2018).

Until now, the nature of inheritance was precisely defined in several early-onset PD (EOPD) forms: autosomal dominantly inherited PD (AD-PD) is associated with the mutations of *LRRK2*, *SNCA*, *VPS35*, *ATXN2*, and *GCH1* genes, autosomal recessive form of PD (AR-PD) with mutations of *PRKN*, *PINK1*, *PARK7*, *ATP13A2*, *PLA2G6*, *FBXO7*, *DNAJC6*, *SPG11*, *SYNJ1*, and *VPS13C* genes (Spatola and Wider, 2014; Lill, 2016; Lunati et al., 2018). Regarding the implication of candidate genes in pathogenesis of PD, the available evidences are often not fully convincing. In fact, further functional studies are needed to clarify their direct role (such as in the case of *DNAJC13*, *TMEM230*, *UCHL1*, *RIC3*, *HTRA2*, *GIGYF2*, *CHCHD2*, *EIF4G1*, *PTRHD1*, *PODXL*, *ABCA7*, and *DCTN1*) (Araki et al., 2014; Nuytemans et al., 2016; Cardona and Perez-Tur, 2017; Puschmann, 2017; Lunati et al., 2018).

Monogenic forms of PD are detected relatively rarely, even in cases where early-onset and/or familial accumulation suggests a strong heritability. In the recent years, the better availability of NGS has made it possible for new candidate genes to be identified in the background of PD. Analysis of genomic data has revealed that rare variants in genes that are linked to PD could provide new biological insight into the complex genetic aetiology of the disease. Genetic testing may play an increasing role, both in the counselling of individual patients and their families with respect to the expected disease course and recurrence risks, and in the stratification of patient groups in clinical trials (Gasser, 2015).

The aim of this study was to analyse the genetic background of EOPD in a Hungarian cohort of patients and to evaluate the clinical utility of different genetic investigations. To maximise the possibility to find potential causative mutations early-onset (25–50 age of onset) and/or positive familiar history were the most important selection criteria. Our cohort was analysed by covering the most frequent PD-associated genes with Sanger sequencing and MLPA analysis. Moreover, we also examined the possible involvement of other genes which may relate with PD by performing NGS experiments. To date, it has been the first comprehensive genetic study on Hungarian patients with PD that exploits the opportunities provided by the NGS method.

## Materials and Methods

### Studied Cohort

Our study involved 142 Hungarian patients, who were diagnosed with early-onset (age of onset: 25–50) Parkinsonism based on Movement Disorder Society (MDS) criteria (bradykinesia in combination with either resting tremor, rigidity, or both) (Postuma et al., 2015). Every case was examined by a board-certified neurologist. Our cohort included 64 females (16 familial and 48 sporadic) and 78 males (17 familial and 61 sporadic), the mean age of onset in the whole cohort was 40.1 ± 6.92 (**Table 1**). In our cohort, a case was labelled as familial if PD was reported among first- or second-degree relatives. In 23 out of 33 familial cases, first-degree relatives were affected. All patients were born in Hungary and descended from Hungarian ancestors. Nonrelated healthy subjects (n = 55, mean AOO = 59.7 ± 17.80) and patients without any neurodegenerative symptoms (n = 82, mean AOO = 45.1 ± 15.47), who had available whole exome data, were screened as controls. In addition, we excluded secondary Parkinsonism caused by environmental factors.

**Table 1:** Hungarian patients with early-onset Parkinsonism.

	Number of sporadic cases (mean AOO ± SD)	Number of familial cases (mean AOO ± SD)
**Male**	61 (41.1 ± 6.6)	17 (37.2 ± 6.93)
**Female**	48 (40.9 ± 6.62)	16 (38.1 ± 7.92)

AOO, age of onset; SD, standard deviation.

Patients and controls were collected from NEPSYBANK of the Institute of Genomic Medicine and Rare Disorders at Semmelweis University (Molnar and Bencsik, 2006). Written informed consent in accordance with the Declaration of Helsinki was signed by patients and control subjects before blood collection and molecular genetic analysis were made. The study was approved by the Hungarian Scientific and Research Ethical Committee. Molecular genetic analysis was accomplished for diagnostic purposes in all investigated patients.

### Molecular Genetic Analysis

DNA was extracted from blood using the QIAamp DNA blood kit, according to the manufacturer’s protocol (QIAgen, Hilden, Germany). The whole coding region and exon/intron boundaries of *PRKN* and *PINK1* and 14 exons of *LRRK2* gene (Nuytemans et al., 2010; Cruts et al., 2012), which contained all of the previously reported pathogenic substitutions, were analysed with Sanger sequencing using ABI Prism 3500 DNA Sequencer (Applied Biosystems, Foster City, USA). The genetic sequence was compared with the human reference genome (*PRKN*: ENST00000366898.5, NM_004562, *PINK1*: ENST00000321556.4, NM_032409, *LRRK2*: ENST00000298910.11, NM_198578) using NCBI’s Blast^®^ application. Exon dosage was pre-typed by using multiplex ligation-dependent probe amplification (MLPA, SALSA MLPA Kit P051-D1 Parkinson; MRC Holland, Amsterdam, Netherlands) according to the protocol. Moreover, the most frequent *LRRK2* G2019S substitutions were screened by the same method.

Altogether, 127 genes (**Supplementary Table 1**), which were previously reported in connection with neurodegenerative diseases, were investigated by NGS. During the selection process of these genes, we focused on which were previously related to PD pathogenesis, with high confidence, or potentially cause overlap neurodegenerative diseases.

DNA samples of 54 probands were analysed by whole exome sequencing (WES, n = 14) and targeted NGS (n = 40). Genomic DNA library preparation was performed using Agilent SureSelectQXT Human All Exon v5 reagents and SureSelectQXT Target Enrichment for Illumina Multiplexed Sequencing (Agilent Technologies, Santa Clara, CA, USA) according to the protocol. Library preparation was followed by NGS using Illumina HiSeq PE Cluster Kit v4 for cluster generation on cBot, HiSeq SBS Kit v4 for sequencing on HiSeq2500 system, and MiSeq Reagent Kit v2 (300-cycles) for sequencing on MiSeq (Illumina, San Diego, CA, USA).

### Bioinformatics Analysis

Variant calling from the NGS data was performed by GATK HaplotypeCaller (version 3.3-0) following the GATK Best Practices Guidelines (Van der Auwera et al., 2013). Variant Call Format (VCF) files were annotated with the SnpEff software (Cingolani et al., 2012) and ClinVar database (Landrum et al., 2016). Variant filtration of exome sequencing data was carried out with VariantAnalyzer software developed by the Budapest University of Technology and Economics. The nature of novel alterations was established according to the American College of Medical Genetics and Genomics (ACMG) guideline (Kearney et al., 2011; Richards et al., 2015). The minor allele frequencies (MAFs) of the variants were taken into consideration, predominantly rare variants defined as their MAF did not exceed 1%. The MAF was assessed by the data of 1,000 Genomes Project (1KG), the Exome sequencing project (ESP), Exome Aggregation Consortium Database, and Genome Aggregation Database (gnomAD v2.1) using non-neuro (non-Finnish) population as a reference.

During the analysis, the next step was the selection of known disease associated variants, then nonsynonymous variants with deleterious prediction scores on the protein function were selected. Nonsynonymous alterations were classified based on the dbNSFP database (Liu et al., 2013)

Loss of function (nonsense, stop loss/stop gain, frameshift, canonical splice) variants and missense variants, which were predicted to be deleterious by multiple lines of computational evidence, were reserved for further analysis. The MAFs of the variants were taken into consideration, predominantly rare variants defined as their MAF did not exceed 1%. The MAF was assessed by the data of 1KG, the ESP, Exome Aggregation Consortium Database, and gnomAD using ALL population as a reference and neither population could have higher MAF than 1%. The variants which met these criteria and were also not present in the control group were considered as damaging and were validated by Sanger sequencing. Where family members were available, segregation analysis was carried out in the proband’s family.

### Statistical Analysis

Odds ratios (ORs) and 95% Confidence Intervals (CI) were calculated to estimate risks using Medcalc software (https://www.medcalc.org/calc/odds_ratio.php). Quantitative variables were described using mean value ± standard deviation (SD).

We analysed the effect of rare variant burden on the age at onset of the disease. Namely, the age of onset of patients with zero (87 patient), one (46 patients), two filtered variants (8 patients) were compared with ANOVA test. As three co-occurring variants were only present in one patient, that patient was excluded from the statistical analysis.

To examine the possibility of oligogenic effect, we performed the following analysis. We calculated variant burden in individuals, who underwent NGS (either targeted panel sequencing or WES), where the same set of genes were assessed, which were covered also by the PD-NGS panel (**Supplementary Table 1**). The following groups were created for the analysis: (1) Control group (in total, 124 individual): this consisted individuals without any disease (21 individual), patient with non-neurological diseases (45 patient), and patients with mitochondrial disorder (58 patient). (2) Non-PD neurodegenerative group (in total, 106 patients). These patients were diagnosed with a neurodegenerative disease, without symptoms of PD (such as hereditary spastic paraparesis, ataxia, amyotrophic lateral sclerosis, and dementia). (3) Patients with PD, who underwent NGS testing (54 patients). We defined variant burden as the total number of filtered variants in every gene represented on the NGS targeted panel (in total, 127 genes). Heterozygous variants were counted as 1 hit, homozygous variants as 2 hits. Summing all the hits in all the 127 genes resulted in the final variant burden. We tested the null hypothesis that the distribution of the individual variant burdens in the three groups (control, PD, non-PD neurodegenerative) came from the same sample with Kruskal-Wallis test. We calculated variant burden with an increasingly stringent filter criteria and tested statistical significance on every level for the three above defined group. The following filter criteria were created. (A) maximum MAF in any population is <1% AND effect of mutation is either loss of function (stop gain, stop loss, frameshift, canonical splice site) or missense. (B) The same filter as (A), but only those missense variants were counted, where multiple lines of computational evidence suggested damaging effect. (C) The same filter as (A), but only those variants were counted, which were present in a core PD gene list. These genes are the union of genes listed in the OMIM database or GeneReviews as associated with Mendelian forms of Parkinsonism, namely, *GBA*, *LRRK2*, *PARK7*, *PINK1*, *PRKN*, *SNCA*, *VPS13C*, *VPS35*, *ATP13A2*, *DNAJC6*, *FBXO7*, *SLC6A3*, *SYNJ1*, *DNAJC13*, *CHCHD2*, *PLA2G6*, *EIF4G1.* (D) The same filter as (C), but only those missense variants were counted, where multiple lines of computational evidence suggested damaging effect.

## Results

In our study, 142 unrelated probands with Parkinsonism were analysed, from which, 33 cases familial aggregation of neurodegenerative disorders were reported. The identified variants were classified into four groups: (1) rare damaging mutations in PD-associated genes, potentially compatible with a monogenic PDs; (2) previously reported genetic risk variants of PD; (3) multiple-hit mechanism impacting the risk of PD; (4) single heterozygous mutations in AR-PD-associated genes.

### Rare Damaging Mutations in PD-Associated Genes, Potentially Compatible With a Monogenic PD

In the *PRKN* gene, exon 7 duplication were detected in four sporadic patients. In case of one sporadic patient, *SNCA* gene duplication was identified. All of the detected CNVs were classified as pathogenic based on ACMG classification guideline. A previously reported, *LRRK2* L1795F pathogenic mutation (Nichols et al., 2007) were detected in a single case (P15). In addition, novel damaging mutations were identified both in *LRRK2* (Y1649S) and *EIF4G1* (M1357T) genes in the P123 and P2 patients, respectively. The L2170W mutation in *DNAJC13* gene was classified as a variant with unknown significance, although Gustavsson et al. in 2015 suggested that this variant increase the genetic risk of PD (Gustavsson et al., 2015). Neither of these variants were detected in our control group and the two *LRRK2* (L1975F and Y1649S) mutations were not presented in gnomAD database. All the identified damaging SNVs were found in patients with familial aggregation. Clinical characteristics of the patients are reported in **Table 2**. Patients with *PRKN* exon 7 duplication, who are not involved in this Table, did not have atypical neurological symptoms. Two patients had equivalent, and two patients had akinetic-rigid type of PD. Mean age at onset in this group of patients was 42.25 ± 5.0 years. The P140 male patient has akinetic-rigid type of PD without any atypical symptoms (AOO = 44).

**Table 2:** Patients with rare substitutions in AD-PD-associated genes.

Patient ID	Form	AOO	Sex	Symptoms	Gene	Variant ID	Zygosity	Clinical significance	ACMG classification	MAF	Patients	Controls	Reference
P15	F	25	f	tremor, slower and altered handwriting, depression, anxiety	*LRRK2*	L1795F c.5385G>T rs111910483	het	P	LP	<0.01	1/142	0/117	(Nichols et al., 2007)
P123	F	34	m	hypomimia, tremor, rigidity, bradykinesia, postural instability, freezing, depression	*LRRK2*	Y1649S c.4946A>C –	het	D	LP	–	1/142	0/117	*
P46	F	30	m	tremor, rigidity, bradykinesia, decreased synkinesis of arms, depression	*DNAJC13*	L2170W c.6509T>G rs140537885	het	D/RF	US	<0.01	2/54	0/117	(Gustavsson et al., 2015)
P2	F	33	f	tremor, decreased synkinesis of arms	*EIF4G1*	M1356T c.4067T>C rs144059151	het	D	US	<0.01	1/54	0/117	*

For minor allele frequency (MAF), we used the gnomAD database non-neuro group (v2.1) of European population (non-Finnish). F, familiar aggregatio; f, female; m, male; LRRK2, leucine rich repeat kinase 2; DNAJC13, DnaJ heat shock protein family (Hsp40) member C13; EIF4G1, eukaryotic translation initiation factor 4 gamma 1; het, heterozygous; P, pathogenic; D, damaging; RF, risk factor; ACMG, American College of Medical Genetics; LP, likely pathogenic; US, uncertain significance; AOO, age of onset; MAF, minor allele frequency. *Firstly reported in this study.

### Previously Reported Genetic Risk Variants of PD

In our cohort, 25 sporadic cases were identified with previously published genetic risk variants in PD genes (22.94% of sporadic cases) (**Table 3**). This rate is approximately two times higher in familiar cases (45.46%), where 15 patients carried risk variants. In this Hungarian population, heterozygous *GBA* mutations, such as H294Q, E365K, T408M, N409S, and L483P variants, were identified in 10 patients (7.04%) and four controls (3.42%). The detected H294Q, L483P, and N409S *GBA* variants were published as pathogenic in biallelic form in GD. The T408M variant based on ClinVar is considered as benign, the E365K as VUS in GD; however, previous studies identified both of them as genetic risk variants for PD (Benitez et al., 2016; Barkhuizen et al., 2017). Moreover, one patient (P18) carried two previously published genetic PD risk variants in *GBA* gene. Among the *GBA* carriers, some patients had distinctive clinical features, such as supranuclear vertical gaze palsy (P25), nonmedication associated hallucinations (P19), depression and anxiety (P13, P18), and early cognitive dysfunction in two patients (P13, P24).

**Table 3:** Previously described genetic risk factors associated with PD.

Patient ID	Gene	Variant ID	Zygosity	MAF	Patients	Control	Sporadic	Familial	Mean AOO	OR	*p*	Reference
P10	*GBA*	L483P c.1448T>C rs421016	het	<0.01	1/54	0/137	–	1	27	7.7	0.21	(Benitez et al., 2016)
P13	*GBA*	H294Q c.882T>G rs367968666	het	<0.01	1/54	0/137	–	1	30	7.7	0.21	(Benitez et al., 2016)
P16, P17	*GBA*	E365K c.1093G>A rs2230288	het	0.01	2/54	1/137	2	–	47	5.2	0.18	(Barkhuizen et al., 2017; Benitez et al., 2016)
P8, P18, P23, P24, P25	*GBA*	T408M c.1223C>T rs75548401	het	<0.01	5/54	2/137	3	2	33.6	6.9	0.02	(Barkhuizen et al., 2017; Benitez et al., 2016)
P18, P19	*GBA*	N409S c.1226A>G rs76763715	het	<0.01	2/54	1/137	2	–	36	5.2	0.18	(Barkhuizen et al., 2017; Benitez et al., 2016)
P26, P31, P43, P104	*LRRK2*	M1646T c.4937T>C rs35303786	het	0.02	4/142	0/137	2	2	43.8	8.9	0.15	(Paisán-Ruiz et al., 2013)
P1, P6-P7, P14, P21-P22, P40-P41, P51, P57-P58, P61, P73-P76, P88, P110, P117, P121, P124	*LRRK2*	S1647T c.4939T>A rs11564148	hom	0.31	21/142	14/137	13	8	41.7	1.5	0.25	(Zheng et al., 2011)
P11, P56, P62, P93	*PINK1*	A340Tc.1018G > Ars3738136	het	0.05	4/142	12/137	3	1	39	0.3	0.04	(Wang et al., 2006)
P1, P28	*PINK1*	G411Sc.1231G > Ars45478900	het	<0.01	2/142	1/137	1	1	36.5	1.9	0.59	(Puschmann et al., 2017)

For minor allele frequency (MAF), we used the gnomAD database non-neuro group (v2.1) of European population (non-Finnish). AOO, age of onset; het, heterozygous; hom, homozygous; GBA, glucosylceramidase beta; LRRK2, leucine rich repeat kinase 2; MAF, minor allele frequency; OR, odds ratio; PINK1, PTEN induced kinase 1; p, probability value.

Segregation studies were successful in three cases: P10 had L483P/c.1448T>C mutation, his father, fraternal grandmother, and her sister carried the same rare variant in the *GBA* gene. The sister has late-onset PD with severe cognitive dysfunction. Other family members did not have any PD signs or symptoms. P13 harbours the H294Q/c.882T>G mutation. His grandfather and the brother of his grandfather had late-onset PD. We were able to analyse his father who has no signs of PD but carries the mutation. P25 patient carries the heterozygous T408M/c.1223C>T mutation. His brother with similar symptoms also carries this heterozygous substitution. The healthy mother has no mutation in the *GBA* gene, and the father died at the age of 41. The father’s sibling also suffered from PD, so we supposed that the brothers inherited the c.1223C>T substitution from their father.

In the *LRRK2* gene, the heterozygous M1646T mutation was found in four patients compared to zero in control group. In 21 patients (14.8%), *LRRK2* S1647T substitution was detected in homozygous form, which were previously suggested to contribute PD risk (Zheng et al., 2011). However, it was also detected in our control group in homozygous form in 14 subjects (10.22%). The *PINK1* A340T polymorphism was identified in four cases among patients and in 12 control subjects. The G411S substitution was also detected in two cases and one control in our cohort.

Among all the detected, previously published genetic risk variants, all of it are associated with an OR > 1 in our cohort; however, we could only confirm two risk variants (*GBA* T408M and *PINK1* A340T), to be significantly more frequent in cases, compared to controls.

### Multiple-Hit Mechanism Impacting the Risk of PD

It is hard to interpret multiple-hit mechanism on an individual level. However, we have observed in nine cases that heterozygous damaging mutations in AR-PD-associated genes coexisted with either a known risk or a rare variant in another PD-associated gene (**Table 4**). The coexistence of the *LRRK2* risk variant and an additional heterozygous missense mutation of *PRKN*, *PINK1*, or *SYNJ1* might be compatible to an oligogenic model. In three cases, *LRRK2* S1647T homozygous risk variant was present simultaneously with heterozygous *PRKN* (C446S), *PINK1* (R501Q), or *SYNJ1* (Q1163E) substitutions, respectively. In the case of P7 patient, we assume that the previously unpublished C446S mutation in *PRKN* gene is probably pathogenic based on the *in silico* prediction scores and segregation analysis. P7’s father, who has later onset PD, was carrying heterozygous *PRKN* C446S and *LRRK2* S1647T mutations and P7’s unaffected mother was carrying heterozygous *LRRK2* S1647T substitution. The *PINK1* R501Q alteration was consistently predicted as damaging. Finally, a rare damaging *SYNJ1* Q1163E substitution was found in a male patient with typical features of PD. In two other cases, the *LRRK2* M1646T alteration was identified with other PD-related mutations, such as *PRKN* heterozygous R234Q substitution with damaging prediction scores in P26 or a novel *DNAJC13* heterozygous D1301V damaging substitution in P43 (**Table 4**). We hypothesised that, if a genetic risk factor is concomitantly present with a rare heterozygous damaging mutation, it results in earlier onset of symptoms, compared to cases with a single PD risk variant.

**Table 4:** Double-hit mechanism impacting the risk of PD: Rare damaging variants and previously described genetic risk factors affecting more than one PD associated genes.

Patient ID	Form	AOO	Sex	Symptoms	Gene	Variant ID	Zygosity	Clinical significance	ACMG classification	MAF	Patients	Controls	Reference
P7	F	43	m	hyposmia, dysarthria, tremor, bradykinesia, postural instability, dysdiadochokinesis, MCI	*PRKN*	C446S c.1336T>A –	het	D	P	–	1/142	0/137	*
					*LRRK2*	S1647T^+^ c.4939T>A rs11564148	hom	RF	–	0.31	21/142	14/137	(Zheng et al., 2011)
P26	F	40	f	tremor, postural instability, disturbed vision, MCI	*PRKN*	R234Q c.701G>A rs144032774	het	D	US	<0.01	1/142	0/137	*
					*LRRK2*	M1646T^+^ c.4937T>C rs35303786	het	RF	–	0.02	4/142	0/137	(Paisán-Ruiz et al., 2013)
P21	F	39	f	rigidity, bradykinesia, postural instability	*PINK1*	R501Q c.1502G>A rs61744200	het	D	US	<0.01	1/142	0/137	(Marongiu et al., 2008)
					*LRRK2*	S1647T^+^ c.4939T>A rs11564148	hom	RF	–	0.31	21/142	14/137	(Zheng et al., 2011)
P22	F	37	m	dysarthria, hypophonia, bradykinesia, tremor, RBD, **medication associated fluctuations with OFF dystonia,** panic attacks, **MCI**	*SYNJ1*	Q1163E c.3487C>G rs768503724	het	D	US	<0.01	1/54	0/137	*
					*LRRK2*	S1647T^+^ c.4939T>A rs11564148	hom	RF	–	0.31	21/142	14/137	(Zheng et al., 2011)
P43	S	38	f	tremor, rigidity, decreased synkinesis of arms	*DNAJC13*	D1301V c.3902A>T rs748182915	het	D/RF	LP	<0.01	1/54	0/137	*
					*LRRK2*	M1646T c.4937T>C rs35303786	het	RF	–	0.02	4/142	0/137	(Paisán-Ruiz et al., 2013)
P18	S	31	m	tremor, bradykinesia, rigidity, frequent freezing, **depression, anxiety**	*GBA*	T408M^+^ c.1223C>T rs75548401	het	RF	–	<0.01	5/54	2/137	(Barkhuizen et al., 2017; Benitez et al., 2016)
					*GBA*	N409S^+^ c.1226A>G rs76763715	het	RF	–	<0.01	2/54	1/137	(Barkhuizen et al., 2017; Benitez et al., 2016)
P20	F	39	m	tremor, rigidity, Parkinsonian gait, anxiety, MCI	*PLA2G6*	V630G c.1889T>G –	het	D	P	–	1/54	0/137	*
					*DNAJC13*	R2115Q c.6344G>A rs770715465	het	D/RF	US	<0.01	1/54	0/137	(Vilariño-Güell et al., 2014)
P4	F	39	f	dysarthria, tremor, postural instability, bradykinesia, rigidity, **orthostatic hypotension, low iron level**, MCI	*C19orf12*	L61* c.182T>G –	het	P	P	–	1/54	0/137	*
					*SYNJ1*	E67A c.200A>C rs199750187	het	D	US	<0.01	1/54	0/137	*
P8	F	39	m	tremor, bradykinesia, dysdiadochokinesis, decreased synkinesis of arms	*DNAJC6*	M133L c.397A>T rs61757223	het	D	US	<0.01	1/54	0/137	*
					*DNAJC13*	L2170W c.6509T>G rs140537885	het	D/RF	US	0.01	2/54	0/137	(Gustavsson et al., 2015)
					*GBA*	T408M^+^ c.1223C>T rs386626586	het	RF	–	<0.01	5/54	2/137	(Barkhuizen et al., 2017; Benitez et al., 2016)

In this table, the coexisting rare damaging variants and previously described genetic risk factors in different PD associated genes are presented. ACMG classification was not applied in case of previously described genetic risk factors. For minor allele frequency (MAF), we used the gnomAD database non-neuro group (v2.1) of European population (non-Finnish). The distinctive phenotypic features are indicated with bold. ACMG, American College of Medical Genetics; AOO, age of onset; C19orf12, chromosome 19 open reading frame 12; D, damaging; DNAJC13, DnaJ heat shock protein family (Hsp40) member C13; DNAJC6, DnaJ heat shock protein family (Hsp40) member C6; F, familial aggregation; f, female; GBA, glucosylceramidase beta; het, heterozygous; hom, homozygous; LP, likely pathogenic; LRRK2, leucine rich repeat kinase 2; m, male; MAF, minor allele frequency; MCI, mild cognitive impairment; P, pathogenic; PINK1, PTEN induced kinase 1; PLA2G6, phospholipase A2 group VI; PRKN, parkin RBR E3 ubiquitin protein ligase; RBD, REM (rapid eye movement) behaviour disorder; RF, risk factor; S, sporadic case; SYNJ1, synaptojanin 1; US, uncertain significance. *Firstly detected in this study. ^+^ Previously identified as genetic risk factor in the refereed publications.

**Figure 1:**
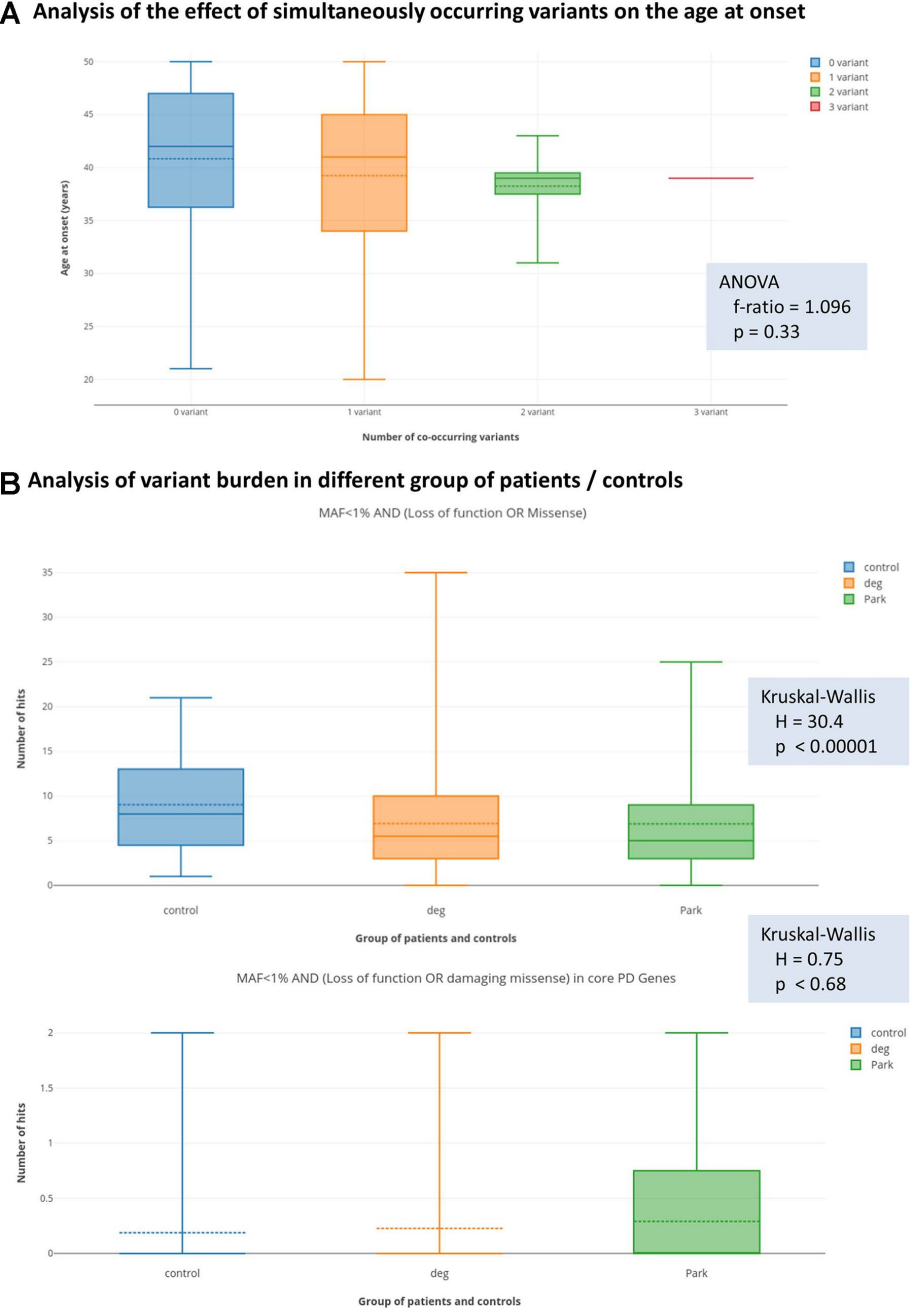
Results of the analysis of oligogenic effects. Panel **(A)** shows the result of analysis coexisting variants (multiple-hits) on the age at onset. The box plots show the distribution of the age at the onset of symptoms if zero, one, two, or three simultaneous variants were present. Solid lines show medians and dashed lines the means. Differences between groups were not significant with ANOVA test. Panel **(B)** shows variant burden in the groups defined at the Methods section. Deg = patients with neurodegenerative diseases, without signs of Parkinsonism. Park = patients with Parkinsonism. We present the results with the least and most stringent filter criteria set. On the upper part of panel **(B)** (less stringent filters), we can see that more variants were present in the control group, which actually reached the level of statistical significance. On the lower part of panel **(B)** (most stringent applied filters), we can see that this difference diminished and even seemed to turn-around; however, in this case, the difference was not significant.

In order to test the hypothesis of oligogenic inheritance pattern, we performed two analyses. Firstly, we tested with ANOVA, whether patients with more than one filtered genetic variant (presented in **Table 4**, in total nine patients) have different distribution of age at onset from those, who harbour only one filtered variants (presented in **Tables 2**, **3**, and **5**, in total 46 patients). We could only observed a trend to have lower mean and median age at onset with increasing number of filtered co-occurring variants; however, this did not reach the level of statistical significance (**Figure 1A**).

**Table 5:** Possibly relevant rare heterozygous variants in AR genes implicated in PD susceptibility.

Patient ID	Form	AOO	Sex	Symptoms	Gene	Variant ID	Zygosity	Clinical significance	ACMG classification	MAF	Patients	Controls	OR	*p*	Reference
P27	S	35	f	rigor, hypokinesis, tremor, incontinence, swallowing difficulties, hyposmia, **low serum CP level**	CP	I898M c.2694T>G -	het	D/RF	LP	–	1/54	0/137	7.7	0.21	*
P36	S	42	f	bradykinesia, postural instability, impaired sense of smell, dysarthria, MCI	*DNAJC6*	F414Y c.1241T>A -	het	D/RF	LP	–	1/54	0/137	7.7	0.21	*
P100	S	42	m	tremor, rigidity, bradykinesia, postural instability	*PRKN*	R275W c823C>T rs34424986	het	P/RF	LP	<0.01	1/142	0/137	2.9	0.65	(Abbas et al., 1999)
P9	S	49	m	**hallucinations, proximal weakness of legs, spasticity, incontinence. MRI: severe white matter lesio**ns	*PLA2G6*	R39W c.115C>T rs763352728	het	D/RF	LP	<0.01	1/54	0/137	7.7	0.21	*

For minor allele frequency (MAF), we used the gnomAD database non-neuro group (v2.1) of European population (non-Finnish). The distinctive phenotypic features are indicated with bold. ACMG, American College of Medical Genetics; AOO, age of onset; CP, ceruloplasmin; D, damaging; DNAJC6, DnaJ heat shock protein family (Hsp40) member C6; F, female; het, heterozygous; LP, likely pathogenic; m, male; MAF, minor allele frequency; OR, odds ratio; P, pathogenic; p: probability value; PLA2G6, phospholipase A2 group VI; PRKN, parkin RBR E3 ubiquitin protein ligase; RF, risk factor; S, sporadic case. *Firstly detected in this study.

Secondly, we tested the hypothesis with the Kruskal-Wallis statistics, that in patients with Parkinsonism, there is a greater variant burden in PD-associated genes compared to non-PD neurodegenerative patients, and control individuals, as described in the Statistics section. Surprisingly, with the most relaxed filter criteria (MAF < 1%, AND loss of function or missense variants), we observed that there is a significantly greater number of variants in the control group (**Figure 1B**) compared to the PD-patient group. However, when we performed the analysis with a stringent filter criterion (MAF < 1% AND loss of function or damaging missense variant in core PD-genes), we observed that this difference diminished, and even seemed to turn around. However, this did not reach the level of statistical significance.

### Single Heterozygous Mutations in AR-PD Associated Genes

In one sporadic case, pathogenic R275W heterozygous mutation was identified in *PRKN* gene, and in three sporadic cases, possible damaging substitution were identified in autosomal recessively inherited genes, such as *DNAJC6* (F414Y), *CP* (I898M), and *PLA2G6* (R396W) (**Table 5**). Notably, AOO is comparably high as it was seen in case where previously described genetic risk factors were solely presented (**Table 3**).

## Discussion

This is the first genetic epidemiology study targeting disease genes of early-onset PD in Hungary, in which several genetic alterations were identified in PD-associated genes. Since the monogenic forms cause only a minority of EOPD, there is a need to identify the most cost-effective diagnostic workup for these patients. Genetic analysis of Hungarian EOPD patients revealed mutations affecting several genes with varying degree of association, such as *DNAJC6*, *DNAJC13*, *EIF4G1*, *GBA*, *LRRK2*, *PRKN*, *PINK1*, *PLA2G6*, *SNCA*, and *SYNJ1*. Studying the functions of their protein products further elucidate the potential role and interplay in the pathomechanism of neurodegeneration, like mitochondrial dysfunctions and impaired autophagy-based protein or organelle degradation pathways, which indeed can lead to development of PD (Ryan et al., 2015).

The possibility to identify more than one rare variant in a PD patient is not uncommon based on our and others observations (Lubbe et al., 2016; Keogh et al., 2018) in the era of NGS. In our cohort, nine cases were presented with potential oligogenic interaction in the background of their symptoms, from which seven cases had a positive family history (**Table 4**). The coexistence of *LRRK2* M1646T or S1647T risk variants (Zheng et al., 2011; Paisán-Ruiz et al., 2013) with further heterozygous rare damaging missense mutations of *PRKN*, *PINK1*, or *SYNJ1* might suggest an oligogenic inheritance. The *LRRK2* risk variants with mutations in AR-PD-associated genes raised the possibility that maybe these explain the earlier age of onset in these patients. Further analysing the modifying effects of these risk variants, especially if they are associated with recessive heterozygous rare damaging mutation, could potentially clarify the PD prognosis. In our cases, oligogenicity was supposed if a patient had more than one variant (either rare damaging or common risk variant) in different PD-associated genes. Although these observations are interesting, it is hard to interpret them on an individual level. In order to support statistically the possibility of an oligogenic effect, we tested two hypotheses. We asked, whether those patients, who simultaneously carry more than one PD-associated variant, have earlier age at onset. We could observe a trend, however, this did not reach statistical significance (**Figure 1A**). The second question we asked was, whether patients, who are diagnosed with PD, more commonly carry rare variants in PD-associated genes compared to other patients with neurodegenerative diseases or controls. Surprisingly, we have observed that more rare variants were detected if a permissive filter criterion was defined, but this difference diminished with more stringent filter criteria. When we applied a rigorous filter criterion, it even seemed that this trend turns around; however, this did not reach statistical significance (**Figure 1B**). Furthermore, we observed a potential trend in our cohort, that those patients with co-occurring variants had a greater chance for being labelled as familial. In summary, we can only raise the suspicion of an oligogenic effect, but probably much larger cohort are needed to prove this unequivocally.

It has to be emphasised, that limitation of statistical comparison, which is not only the small number of cases (as only 54 of our patients were analysed by NGS; thus, it is possible that they may harbour additional variants in genes that were not covered). But, the different penetrance of the coexisting genetic risk factors (e.g., the low penetrance *LRRK2* S1647T versus a pathogenic *GBA* H294Q mutation), and these tests need to be repeated in larger samples.

The MLPA analysis identified five cases with pathogenic mutation from the 142 EOPD patients (3.5%), while Sanger sequencing revealed potential damaging mutation of *LRRK2* in two cases and risk variants in *PINK1* and *LRRK2* genes in 28 cases (19.7%). Co-occurring variants were detected in three patients (2.1%). A heterozygous *PRKN* mutation was detected in a single case (0.7%). The NGS of 54 patients revealed mutations affecting several other PD-associated genes (*CP*, *C19ORF12*, *DNAJC6*, *DNAJC13*, *EIF4G1*, *GBA*, *PLA2G6*, *SYNJ1*). Two patients carried heterozygous variants in AD-PD genes (3.7%; LRRK2, DNAJC13, EIF4G1), while 10 patients carried *GBA* mutations (18.5%). Six patients were identified with potential oligogenic interactions with the coexistence of genetic risk factor or heterozygous rare variants in PD-associated genes (11.1%). Three patients carried a single heterozygous mutation in an AR-PD gene (5.6%). Altogether, we conclude that whole exome sequencing potentially increased the number of relevant findings.

Among the 109 sporadic cases, five patients have CNVs in *PRKN* or *SNCA* genes. Deletions and duplications/triplications of one exon or exon groups account for a significant proportion of mutations in the *PRKN* gene (Shulskaya et al., 2017). In a Dutch cohort, the exon 7 duplication was suggested to be a founder mutation based on haplotype analysis and shared similar breakpoints (Elfferich et al., 2011). The importance of CNVs in *PRKN* gene was supported by our results as well, since 23.5% of our solved cases (consistent either to oligogenic or AD inheritance) harboured *PRKN* exon rearrangement, which were presumed to be more deleterious, because they caused altered *PRKN* protein function by reorganising the protein structure (Pankratz et al., 2011). These findings suggest that examination of CNVs in most frequently affected genes may be cost-effective in routine clinical practice for diagnosing PD and could be offered as a first step if the genetic workup is considered.

Moreover, PD susceptibility factors in *GBA*, *LRRK2*, and *PINK1* genes were relatively common in our cohort. These variants can be important from clinical point of view since some of them may serve as an important factor for stratification for clinical trials, such as *GBA*, others may contribute as modifiers to the complex aetiology of the disease. Most of the potential genetic risk factors of PD have been detected in genes which were previously associated with Mendelian inherited PD. According to the hypothesis of Sparato et al. (2017), genes harbouring both causal mutations for Mendelian disorders and risk variants for complex diseases may have high functional relevance in the pathogenesis (Spataro et al., 2017). In our cohort, we observed a similar frequency of genetic risk variants as reported in the literature (Karimi-Moghadam et al., 2018). In the future, potential therapeutic intervention could be planned based on some genetic risk variants, similarly to glucosylceramide synthase (GCS) inhibition of *GBA* associated PD (Sardi et al., 2017).

In our study, we identified four rare damaging heterozygous mutations in AR genes (**Table 5**), which suggested a potential role in the PD pathogenesis (Makovac et al., 2016; Ferese et al., 2018). Other studies suggested that approximately 5%–10% of sporadic EOPD patients carried heterozygous mutation in AR-PD-associated genes (Periquet et al., 2003; Clark et al., 2006). In our cohort, similar distribution (7.21%) was detected. Furthermore, it remains a question of debate how heterozygous alteration in recessive genes could contribute to the development of EOPD. Some results indicated that heterozygous mutations may simply result in subclinical cellular dysfunction or predispose PD (West et al., 2002; Periquet et al., 2003; Hedrich et al., 2004). Additionally, single heterozygous mutations in *PRKN* and *PINK1* genes are considered minor genetic risk factors for developing PD and single heterozygous substitutions in *PRKN* gene have been described in sporadic, late-onset PD (Spatola and Wider, 2014). Furthermore, the previously described mutations can cause malfunction in the metabolism of dopamine in the striatum and brain network changes, leading to cognitive impairment (Hilker et al., 2001; Khan et al., 2002; Benbunan et al., 2004; Ricciardi et al., 2014; Makovac et al., 2016). Moreover, other authors suggested that heterozygous mutations in *PLA2G6* may also contribute to the susceptibility for developing PD (Ferese et al., 2018), as some *PLA2G6* heterozygous mutations were presented in PD patients (Bower et al., 2011; Lu et al., 2012). We assume that the I898M damaging mutation in the *CP* gene, which plays an important role in the iron and copper metabolism in the brain (Zhao et al., 2018), could intensify the risk of developing PD. These four patients in **Table 5** with a mean AOO (42 ± 5.72 years) is rather like the sporadic patients with a single genetic risk variant presented in **Table 3** with a mean AAO (40 ± 6.94 years) than the sporadic patients with potential oligogenic background (presented in **Table 4** with a mean AOO 34.5 ± 4.95 years). Examining mutations in AR-PD-associated genes may be essential to further specify the contribution of heterozygous mutations in patients with EOPD. Additional studies need to further elucidate whether these variants are potential AD-PD-inducing mutations, or they act as risk factor with coexisting genetic susceptibility and environmental effects. Exploring the function of monoallelic alterations in AR-PD may be crucial for possible future use of genetic data for improvement of genetic counselling in individualised settings.

**Figure 2:**
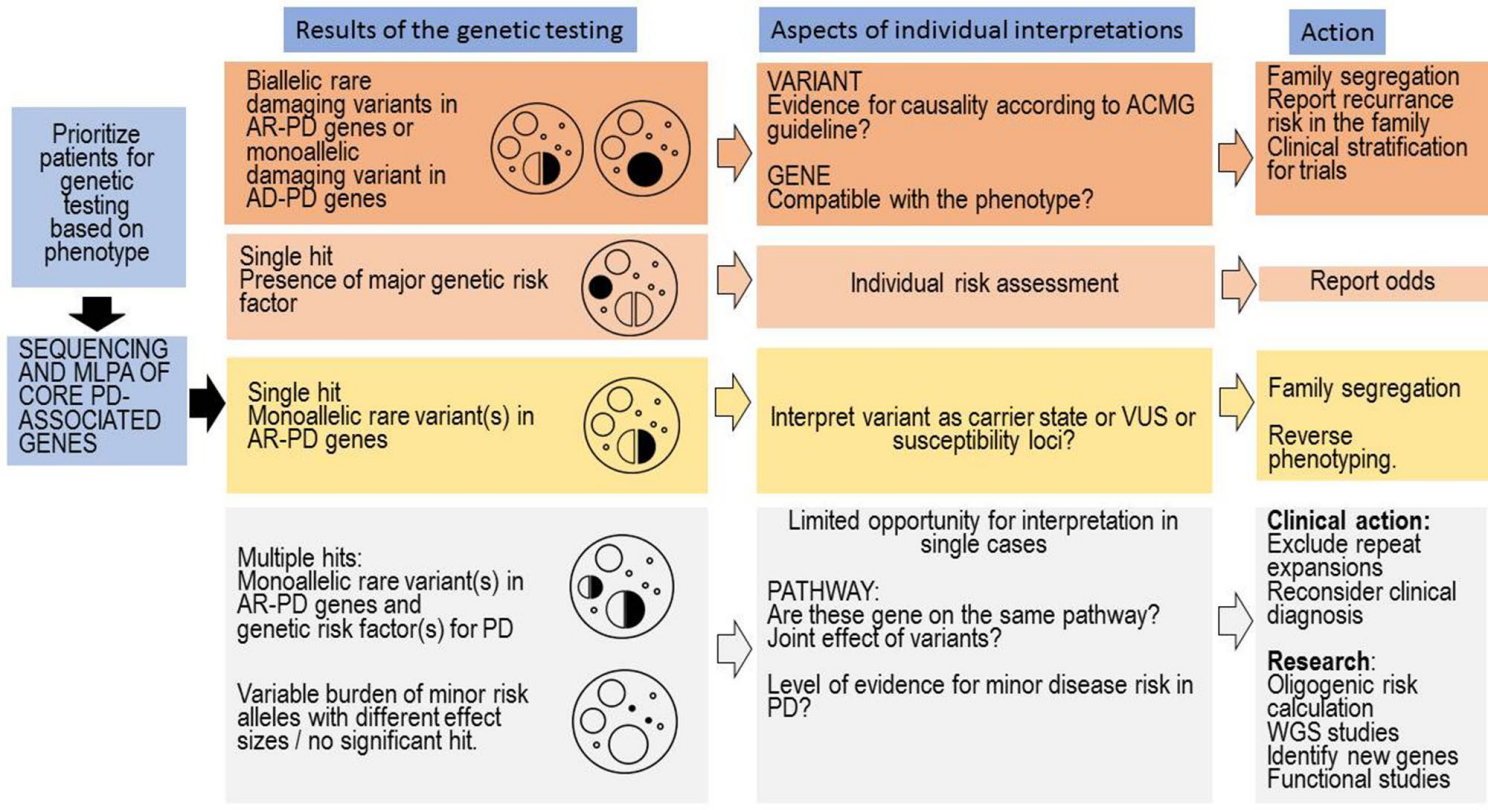
Clinical interpretation of genetic test results and tasks associated with the genetic counselling. The first column represents the most common situations emerging after genetic testing in PD. On the top, we included the more easily interpretable findings, and on the bottom, the commonly emerging challenges. On every level, many aspects of the genetic findings have to be interpreted by the genetic counsellor, and we only included the major questions associated with a level. At the last column, we included important tasks of the genetic counsellors associated with a given level; however, naturally in every level, a complex approach is needed.

The genetic testing in PD is complex and it depends on whether testing is being requested by someone who has PD or someone who has positive family history of PD, without symptoms of PD. The complexity is coming from the fact that several genes and risk factors are associated with PD. In many patients with PD, even if they have a family history for PD, the disease-causing abnormal genes cannot be detected. Notably, the genetic counselling is specially challenging in those cases where (1) several genetic risk factors or (2) a genetic risk factor and a heterozygous carrier status of a pathogenic or likely pathogenic mutation is coexisting (**Figure 2**). The co-segregation analysis of these coexisting rare variants can facilitate understanding the role of these variants. The *GBA* variants may have particular importance, based on the published data, it seems that, even in heterozygous status the variant, which has a more severe effect to the protein, has greater impact on the disease onset and progression (Zhang et al., 2018). The clinicians should differentiate in counselling where a healthy person without any family history request the counselling asking the risk scores of different genetic disorders (among them PD), or a patient having PD asking the recurrence risk in the next generation, or if the genetic results of the relatives of a PD patients has to be interpreted. Nevertheless, the situation is complicated if the person has monoallelic deleterious rare variant in AR inherited PD. In cases having coexisting monoallelic rare pathogenic variant and alteration(s) in genes which could potentially increase PD susceptibility or having more than one rare variant in those genes the risk of disease development is greater. Furthermore, it is important to highlight those further gene-gene interactions and environmental factors also influence the manifestation of the genetic background. In some cases, potential disease-causing mutation in previously PD-associated gene will not ensure the occurrence of PD symptoms during the life of the patient due to the reduced penetrance. Additionally, it is crucial that the genetic counsellor must be familiar with all those distinctive phenotypic features which can be associated with the different PD-associated genes (e.g., *GBA*, *DNAJC6*, *SYNJ1*) since in some cases with likely pathogenic variants or VUS in PD-associated genes, the reverse phenotyping is an important task (**Figure 2**). In our cohort, not only *GBA* PD cases had distinctive clinical features, but a patient with heterozygous *PLA2G6* variant (P9) had hallucinations, proximal weakness in the lower extremity, spasticity, urinary incontinence (**Table 5**). MRI showed severe white matter lesions. Early cognitive dysfunction was present in a patient (P22), who carried heterozygous variants in *SYNJ1* and *LRRK2* (**Table 4**). This patient also had early and severe medication associated fluctuations with frequent OFF dystonia. Orthostatic hypotension was early and severe in a patient with heterozygous *C19orf12* variant (P4) (**Table 4**). In a patient, who carried a heterozygous *CP* variant (P27) had low serum ceruloplasmin level. To collect all this type of phenotypic observations are very important for further understanding the genotype-phenotype correlations in PD.

To identify the genetic background is important not only in family planning but there is an increasing need in the era of the personalised medicine to stratify the patient based on their genetic background. There are clinical trials, which are focusing on the treatment of patients with certain genetic alterations, such as heterozygous *GBA* variants. Ideally, to reach personalised therapeutic interventions in the future, a systems biology approach would be optimal analysing multidimensional clinical and genetic/genomic risk and progression profile.

In summary, our study further supports the hypothesis that alterations in Mendelian PD-associated genes may act as a genetic risk factor for the sporadic form and even a heterozygous mutation in AR-PD genes could contribute to the disease susceptibility. With better availability of NGS technologies for clinical assessment, genetic testing is getting more important tool in the clinical practice and research of the EOPD. Application of NGS can effectively identify coexisting pathogenic rare variants and genetic susceptibility factors which could have significant role in understanding the complex pathogenesis of PD. The simultaneous analysis of neurodegeneration-associated genes could support both the diagnostic workup and the interpretation of clinically distinct phenocopies associated with certain single or coexisting genetic alterations and the spectrum of the neurodegenerative disorders. Due to the complexity of the disease and improving availability of NGS technologies, genetic counselling is getting more challenging by the increasing number of PD-associated rare variants. Thus, the clinical geneticist should be prepared for the genetic counselling of patients with coexisting disease-causing mutations and susceptibility factors. In the near future, coexistence of major disease-causing mutation and minor susceptibility factors could be interpreted at the level of individual.

## Data Availability Statement

The raw data supporting the conclusions of this manuscript will be made available by the authors, without undue reservation, to any qualified researcher.

## Ethics Statement

This study was carried out in accordance with the recommendations of Hungarian Scientific and Research Ethical Committee (37/2014 TUKEB) with written informed consent from all subjects. All subjects gave written informed consent in accordance with the Declaration of Helsinki. The protocol was approved by the Hungarian Scientific and Research Ethical Committee.

## Author Contributions

AI and MM conceived and designed the study. ZG, PB, PK and MM acquired and analyzed the clinical data. AI, AGá, DC and RB performed the genetic data. AI, AGé and VM performed the bioinformatics analysis and interpreted the genetic data. AI wrote the draft of the manuscript and ZG, PB, DC, AGá, VM, AGé, PK and MM provided critical comments on the draft of the manuscript. All authors read and approved the final version of the manuscript.

## Funding

This study was supported from Research and Technology Innovation Fund by the Hungarian National Brain Research Program (KTIA_NAP_2017-1.2.1-NKP-2017-00002) and from Semmelweis University by “The development of scientific laboratories in medicine, health sciences and pharmacy” (EFOP-3.6.3-VEKOP-16-2017-00009). The Variant Analyzer software development was supported by the Hungarian Scientific Research Fund (OTKA K-112915).

## Conflict of Interest

The authors declare that the research was conducted in the absence of any commercial or financial relationships that could be construed as a potential conflict of interest.

## Abbreviations

ACMG, American College of Medical Genetics; AOO, age of onset; D, damaging; DNAJC13, DnaJ heat shock protein family (Hsp40) member C13; EIF4G1, eukaryotic translation initiation factor 4 gamma 1; F, familiar aggregation; f, female; het, heterozygous; LP, likely pathogenic; LRRK2, leucine rich repeat kinase 2; m, male; MAF, minor allele frequency; P, pathogenic; RF, risk factor; US, uncertain significance. *Firstly reported in this study.
