# Supplementary material for: The Role of Genetic Testing in the Clinical Practice and Research of Early-Onset Parkinsonian Disorders in a Hungarian Cohort: Increasing Challenge in Genetic Counselling, Improving Chances in Stratification for Clinical Trials
Source: Front Genet. 2019 Oct 31;10:1061. doi: 10.3389/fgene.2019.01061 (PMC6837163; doi:10.3389/fgene.2019.01061)
Supplement: Supplementary file 1 [file Table_1.docx]

**Supplementary Table 1. List of genes which were investigated by next generation sequencing.**

| **Gene symbol** | **Gene name** |
| --- | --- |
| ABCA7 | ATP binding cassette subfamily A member 7 |
| ADAM10 | ADAM metallopeptidase domain 10 |
| ADORA1 | adenosine A1 receptor |
| AKT1 | AKT serine/threonine kinase 1 |
| ALS2 | alsin Rho guanine nucleotide exchange factor ALS2 |
| ANG | angiogenin |
| APOE | apolipoprotein E |
| APP | amyloid beta precursor protein |
| ATM | ATM serine/threonine kinase |
| ATP13A2 | ATPase cation transporting 13A2 |
| ATP1A3 | ATPase Na+/K+ transporting subunit alpha 3 |
| ATP6AP2 | ATPase H+ transporting accessory protein 2 |
| ATXN2 | ataxin 2 |
| BACE1 | beta-secretase 1 |
| BIN1 | bridging integrator 1 |
| C19orf12 | chromosome 19 open reading frame 12 |
| C9orf72 | chromosome 9 open reading frame 72 |
| CD2AP | CD2 associated protein |
| CD33 | CD33 molecule |
| CFL1 | cofilin 1 |
| CHCHD10 | coiled-coil-helix-coiled-coil-helix domain containing 10 |
| CHCHD2 | coiled-coil-helix-coiled-coil-helix domain containing 2 |
| CHMP2B | charged multivesicular body protein 2B |
| CLU | clusterin |
| CP | ceruloplasmin |
| CR1 | complement C3b/C4b receptor 1 |
| CTSD | cathepsin D |
| CSF1R | colony stimulating factor 1 receptor |
| CST3 | cystatin C |
| DCAF17 | DDB1 and CUL4 associated factor 17 |
| DCC | DCC netrin 1 receptor |
| DCTN1 | dynactin subunit 1 |
| DNAJC13 | DnaJ heat shock protein family (Hsp40) member C13 |
| DNAJC5 | DnaJ heat shock protein family (Hsp40) member C5 |
| DNAJC6 | DnaJ heat shock protein family (Hsp40) member C6 |
| DNMT1 | DNA methyltransferase 1 |
| EIF4G1 | eukaryotic translation initiation factor 4 gamma 1 |
| ELAVL1 | ELAV like RNA binding protein 1 |
| EPHA1 | EPH receptor A1 |
| EPHA4 | EPH receptor A4 |
| ERBB4 | erb-b2 receptor tyrosine kinase 4 |
| FBXO7 | F-box protein 7 |
| FIG4 | FIG4 phosphoinositide 5-phosphatase |
| FTL | ferritin light chain |
| FUS | FUS RNA binding protein |
| GBA | glucosylceramidase beta |
| GCH1 | GTP cyclohydrolase 1 |
| GIGYF2 | GRB10 interacting GYF protein 2 |
| GRN | granulin precursor |
| HNRNPA1 | heterogeneous nuclear ribonucleoprotein A1 |
| HTRA2 | HtrA serine peptidase 2 |
| LRRK2 | leucine rich repeat kinase 2 |
| MAPT | microtubule associated protein tau |
| MARK2 | microtubule affinity regulating kinase 2 |
| MS4A4E | membrane spanning 4-domains A4E |
| MS4A6A | membrane spanning 4-domains A6A |
| NOTCH3 | notch receptor 3 |
| OPTN | optineurin |
| PANK2 | pantothenate kinase 2 |
| PARK2 | parkin RBR E3 ubiquitin protein ligase |
| PARK7 | Parkinsonism associated deglycase |
| PICALM | phosphatidylinositol binding clathrin assembly protein |
| PINK1 | PTEN induced kinase 1 |
| PLA2G6 | phospholipase A2 group VI |
| POLG | DNA polymerase gamma, catalytic subunit |
| PRKRA | protein activator of interferon induced protein kinase |
| PRNP | prion protein |
| PSAP | prosaposin |
| PSEN1 | presenilin 1 |
| PSEN2 | presenilin 2 |
| PTK2B | protein tyrosine kinase 2 beta |
| RAD51 | RAD51 recombinase |
| SLC30A10 | solute carrier family 30 member 10 |
| SLC6A3 | solute carrier family 6 member 3 |
| SNCA | synuclein alpha |
| SNCB | synuclein beta |
| SOD1 | superoxide dismutase 1 |
| SORL1 | sortilin related receptor 1 |
| SPG11 | SPG11 vesicle trafficking associated, spatacsin |
| SPR | sepiapterin reductase |
| SQSTM1 | sequestosome 1 |
| SYNJ1 | synaptojanin 1 |
| TAF1 | TATA-box binding protein associated factor 1 |
| TARDBP | TAR DNA binding protein |
| TBK1 | TANK binding kinase 1 |
| TBP | TATA-box binding protein |
| TH | tyrosine hydroxylase |
| TMEM106B | transmembrane protein 106B |
| TMEM230 | transmembrane protein 230 |
| TOMM40 | translocase of outer mitochondrial membrane 40 |
| TOR1A | torsin family 1 member A |
| TPP1 | tripeptidyl peptidase 1 |
| TREM2 | triggering receptor expressed on myeloid cells 2 |
| TYROBP | TYRO protein tyrosine kinase binding protein |
| UBE3A | ubiquitin protein ligase E3A |
| UCHL1 | ubiquitin C-terminal hydrolase L1 |
| VAPB | VAMP associated protein B and C |
| VCP | valosin containing protein |
| VPS13C | vacuolar protein sorting 13 homolog C |
| VPS35 | VPS35 retromer complex component |
| WDR45 | WD repeat domain 45 |
